# Dimensions of Social Capital for Supporting Informal Carers of Patients With Advanced Chronic Illness in the Community: A Concept Mapping Study

**DOI:** 10.1111/hex.70410

**Published:** 2025-09-26

**Authors:** Marques Shek Nam Ng, Winnie Kwok Wei So, Kai Chow Choi, Wallace Chi Ho Chan, Helen Yue Lai Chan, Carmen Wing Han Chan

**Affiliations:** ^1^ The Nethersole School of Nursing The Chinese University of Hong Kong Hong Kong China; ^2^ Department of Social Work, Education and Community Wellbeing Northumbria University Newcastle upon Tyne UK

**Keywords:** advanced chronic illness, community, concept mapping, informal carers, social capital

## Abstract

**Introduction:**

Social capital, defined as social interactions that increase the productivity of a community, can help address the health needs of the community. Although evidence suggests that carers of patients with advanced chronic illnesses experience a high caregiving burden and require community support, the specific dimensions of social capital they need remain unclear.

**Methods:**

This concept mapping study was conducted between April 2021 and July 2023. In total, 98 stakeholders, including 25 carers, 25 patients, 24 professionals and 24 community members, were recruited through purposive and snowball sampling. The research team initially conducted semi‐structured interviews to brainstorm and collect important statements related to the study objectives. Subsequently, the participants were invited to sort and rate these statements. The responses from the rating and sorting tasks were analysed using multidimensional scaling and hierarchical cluster analysis. The importance ratings and demographic backgrounds were summarised with descriptive statistics.

**Results:**

Five clusters emerged from the analysis: carers' attributes, carers' networks, carers and service providers, carers and the local community, and carers and society. Among these clusters, the participants deemed healthcare and social services the most important, followed by positive interactions with the care recipient, a sense of responsibility, and readiness to accept support. They also valued the support received from family members, friends, neighbours, other carers and reliable communication channels. In addition, inclusive public spaces and flexible working arrangements were considered valuable for providing community support to the carers of patients with chronic illnesses in the community.

**Conclusion:**

Despite the many challenges faced by informal carers of patients with advanced chronic illnesses, social capital can be leveraged to address these issues. The dimensions of social capital identified in this study can serve as a framework for developing social care programmes and policies to support informal carers.

**Patient or Public Contribution:**

Patients, informal carers, professionals and community members involved in the care of patients with advanced chronic illnesses participated in this study.

## 1 Introduction

Social capital refers to social interactions that increase the productivity of a community. These interactions occur between individuals and various social groups, such as neighbourhoods, workplaces, extended families, academic institutions and religious organisations within the community. Social capital encompasses qualities that enhance societal efficiency and promote cooperation by facilitating coordinated actions rather than isolated individual efforts [1]. Social capital, which represents a community's internal strength, can be conceptualised in various forms, such as structural capital, including social networks, and cognitive capital, which comprises invisible elements (e.g., trust). Some other forms connect individuals and social groups within (bonding capital) and across (bridging capital) social levels [2]. Sociologists and economists introduced the concept of social capital in the 1990s to emphasise the value of social interactions. It provides a lens to understand how social interactions can facilitate the functioning of individuals or groups within the community, leading to better outcomes. Recent studies have assessed social capital in various populations, such as patients with specific chronic diseases or those belonging to particular social groups [3, 4]. Although the concept is still being explored, evidence suggests that social capital can promote community health [5]. Mobilising social capital can address the health needs of the community in a sustainable manner. However, ongoing research points to a lack of clarity in the concept, indicating that specific forms of social capital remain to be identified [6].

Evidence suggests that social interactions play a significant role in facilitating informal caregiving. Carers with supportive family, friends and healthcare professionals typically have more tolerable experiences than those who are either socially isolated or routinely engage with an uncooperative social network [7]. An informal carer is someone who consistently provides assistance, support or care to a person they share a personal connection with, often over an extended period and beyond their designated responsibilities [8]. This care is typically unpaid and falls beyond the purview of a structured care system. Informal carers can be close friends, neighbours, spouses, parents, children or relatives. They often engage in informal relationships with care recipients by providing them with daily care. Because of the informal nature of these relationships, these carers dedicate considerably more time to caregiving than paid care workers [9]. In the United States, informal carers provided an estimated 36 billion hours of care in 2021, equivalent to an economic value of USD 600 billion [10].

Caregiving is a challenging responsibility. The multidimensional nature of challenges encountered by the informal carers of patients with chronic illness has been documented in the literature. Fu et al. [11] reported that informal carers often experience depression, stress, increased risk of heart disease, and an overall decline in their health. These challenges arise from their roles, which include disease monitoring, financial provision and psychological support. Given the time and effort dedicated to caregiving, informal carers often have limited capacity for other roles, such as those within their families and careers. As a result, they are often isolated from other individuals or social groups in the community, resulting in a reduction in their investment in social capital [12].

Some informal carers of patients with advanced chronic illnesses, such as chronic obstructive pulmonary disease (COPD), chronic kidney disease (CKD) and heart failure (HF), experience challenges in caregiving, particularly in disease management. In addition to providing daily care, carers need to assist with lifestyle modifications, oversee health monitoring, ensure adherence to medication regimens, and occasionally manage complex medical treatments (e.g., oxygen therapy and dialysis therapy) [13]. However, the trajectory of these chronic illnesses often involves prolonged and fluctuating periods with frequent episodes of exacerbation. Carers are responsible for providing sustained care while managing these health crises, resulting in a high caregiving burden and a need for additional support.

Given the challenging circumstances faced by informal carers, especially those caring for patients with advanced chronic illnesses, support at the community level is crucial to facilitate their caregiving roles. Conventional forms of carer support, including carer training and respite care, are provided in various settings globally [10]. However, with an ageing population and a rising prevalence of chronic illnesses, these supportive services, usually operated by the government or non‐governmental organisations (NGOs), cannot meet the increasing demand. Activating and mobilising community resources might provide more sustainable sources of support. From a social capital perspective, interactions between individuals and social groups can support carers in fulfilling their social roles [9].

In addition to the conventional methods of supporting informal carers, including respite care, training, counselling and financial aid, some countries have adopted social initiatives. For instance, a long‐term care insurance system was established in Japan to fund home‐care services [14]. The United Kingdom has adopted a co‐production approach to organise stakeholders and create innovative solutions to reduce the burden of informal carers [15]. This initiative involves asset mapping, which facilitates a comprehensive understanding of community assets that can be leveraged to develop interventions for supporting informal carers.

In Hong Kong, more than 248,000 individuals (about 3.4% of the total population) are responsible for the care of patients with chronic diseases in their households, with 68.6% serving as informal carers [16]. These informal carers in Hong Kong perceive a significant burden associated with their caregiving role and specific challenges with family caregiving [17]. Given the limited knowledge in Hong Kong regarding interventions to support the informal carers of patients with chronic illnesses, it is crucial to identify resources that can be used to support these individuals. Therefore, this study aimed to explore the insights of various stakeholders to gain a comprehensive understanding of the dimensions of social capital that can support the informal carers of patients with advanced chronic illnesses within the community in Hong Kong. In addition, this study aimed to shed light on stakeholders' perceptions of the importance of each identified dimension.

## 2 Materials and Methods

### 2.1 Design

This concept mapping study was conducted between April 2021 and July 2023 following the methodology suggested by Trochim [18]. Concept mapping is a structured procedure centred on a particular subject or construct, involving the participation of multiple individuals and generating an interpretable graphical representation of their ideas, concepts and the connections between them. Concepts related to the studied issue are gathered using qualitative methods and then sorted and visualised using quantitative methods. The concept mapping process, as proposed by Trochim [18], consists of six stages: (1) preparation, (2) statement generation, (3) statement structuring, (4) statement representation, (5) map interpretation and (6) map utilisation. This structured mixed‐method process provides a robust approach to understanding complex social issues [19, 20, 21] and developing healthcare services [22, 23]. This paper presents the findings from the Phase II study, focusing on the structuring and representation of statements and the interpretation of the resulting map.

### 2.2 Inclusion and Exclusion Criteria

A diverse and representative sample of community stakeholders was recruited to generate a comprehensive picture. Four stakeholder groups were identified: (1) patients, (2) informal carers, (3) professionals and (4) community members. Detailed information on selection criteria and recruitment processes was provided in our previous publication [24, 25].

In this study, we included adult patients who were diagnosed with Global Initiative for Chronic Obstructive Lung Disease stage 3/4 COPD, Kidney Disease: Improving Global Outcomes stage 4/5 CKD, or New York Heart Association class III/VI HF. Informal carers were significant others responsible for caring for a patient with any of the aforementioned conditions. Professionals were qualified health and social care practitioners with relevant clinical experience. Community members were experts identified by the research team who possessed knowledge related to the research topic.

The sample size was estimated to ensure an adequate number of participants for the generation and structuring phases [18]. A review [26] reported that previous concept mapping studies included a mean of 71 participants in the structuring phase (i.e., rating and sorting procedures). Considering an average attrition rate of 38.9% and to maintain a balance in the numbers of different stakeholders, we initially aimed to recruit 120 participants (30 in each stakeholder group). However, because of the Covid‐19 pandemic, recruitment was terminated in May 2022. Finally, 98 participants (25 patients, 25 carers, 24 professionals and 24 community members) were recruited.

### 2.3 Data Collection

The participants were recruited in the previous stage (preparation and generation of statements). They were identified from the collaborating health and social care units (patients, carers and professionals) and the research team's networks (community members). A research assistant (RA) invited these individuals to participate. After obtaining informed consent, we scheduled individual interviews for the Phase I study (i.e., preparation and generation of statements).

In this Phase II study, the same RA invited participants to complete an online survey by sending the link through instant messages. Participants who reported difficulty in completing the online survey were interviewed by the RA in person. During these in‐person interviews, the rating and sorting procedures were conducted using paper forms and sorting cards. All participants received a cash coupon as an incentive upon completing the Phase II study.

### 2.4 Instruments

To conduct the rating and sorting procedures, the research team designed a survey based on the concept mapping methodology and our qualitative findings. In the Phase I study, 31 statements related to social capital were generated, with their corresponding forms of social capital presented in Table 1. The participants were initially asked to rate the importance of each statement on a 7‐point scale (1 = not important to 7 = very important). In the second part, they sorted these statements into groups based on their similarity. Participants were provided with detailed examples when they found the statements ambiguous or overlapping [25]. In addition, the background characteristics of the participants were collected during the Phase I study using a demographic form.

**Table 1:** List of generated statements and corresponding forms of social capital.

Statements	Forms of social capital
1. Home‐based support	Bridging capital
2. Need‐oriented service design	Bridging capital
3. Discharge support	Bridging capital
4. Mutual support from neighbours	Bonding capital
5. Referral by social workers	Bridging capital
6. Reaching out proactively	Bridging capital
7. Researching carer situations	Linking capital
8. Increasing resources for carer services	Linking capital
9. Community‐based healthcare	Bridging capital
10. Caregiving information	Bridging capital
11. Support from other family members	Bonding capital
12. Sense of responsibility	Bonding capital
13. Venting space	Bridging capital
14. Mutual support of carers	Bonding capital
15. Reliable, effective communication channels	Bridging capital
16. Coordinating carer services	Linking capital
17. Optimising treatments for patients	Bridging capital
18. Flexible working arrangements	Linking capital
19. Financial and material assistance	Bridging capital
20. Social networks of carers	Bonding capital
21. Inclusive public spaces	Bridging capital
22. Healthcare professionals' attitudes	Bridging capital
23. Respite care	Bridging capital
24. Timely transition to institutional care	Bridging capital
25. Trained volunteer workforce	Bonding capital
26. Healthcare professionals' knowledge of social care	Bridging capital
27. Advocacy work	Bridging capital
28. Patient transport services	Bridging capital
29. Carer‐centred care approach	Bridging capital
30. Interactions between patients and carers	Bonding capital
31. Readiness for accepting support	Bonding capital

### 2.5 Data Analysis

The responses to the rating and sorting tasks were analysed using multidimensional scaling (MDS) and hierarchical cluster analysis (HCA) [18]. The similarity between any two statements was quantified based on the average similarity sorted by the participants. The average similarity score of any two statements was calculated using the following formula: number of participants who sorted the two statements into the same group/total number of participants, which ranges from 0 to 1. A proximity matrix (i.e., 31 statements × 31 statements) was constructed by entering the average similarity scores. Subsequently, MDS was applied to analyse the proximity matrix and models with up to three dimensions were then developed. A maximum of three dimensions was considered for ease of interpretation [18]. The number of dimensions in the final solution was determined using a scree plot of Kruskal's stress against dimensionality. Kruskal's stress value is a measure of goodness‐of‐fit for MDS models, ranging from 1 (indicating the poorest fit) to 0 (representing a perfect fit). Additionally, the R‐squared (RSQ) statistic is often reported to evaluate the final model fit. RSQ represents the squared correlation coefficient of the input distances with the scaled distances using the MDS coordinates, indicating the proportion of variance explained by the solution. A Kruskal's stress value of ≤ 0.285 and an RSQ of ≥ 0.6 were considered acceptable for achieving the balance between model fitness and variance [27].

A point map was developed using this final MDS solution to visualise the relative similarities among statements. Although the distance between any two points on this map indicated the relative similarity between the corresponding statements, HCA was performed to group points on the point map into clusters using Ward's algorithm [28]. The number of clusters in the final HCA solution was determined using the dendrogram and an iterative interpretation process on the cluster contents by the research team, as recommended by Trochim [18]. The research team labelled each cluster based on the included statements. The importance ratings and demographic backgrounds were summarised using appropriate descriptive statistics, such as frequencies, percentages, means and standard deviations (SDs). All statistical analyses were performed using IBM SPSS 26.0 software package (IBM Corp., Armonk, New York, the United States).

### 2.6 Ethical Considerations

This study was approved by The Survey and Behavioural Research Ethics Committee of The Chinese University of Hong Kong (Reference Number: SBRE‐19‐715). Although the participants had already provided their consent during the Phase I study, informal consent was obtained again from the participants before data collection. The participants were reassured of their right to withdraw from the study at any point. According to institutional policies, all collected data were kept confidential and securely stored in locked drawers and on encrypted devices. In compliance with the requirements set by the funding body, anonymised data from the Phase II study will be made publicly available 3 years after the study's completion. Members of the public can access the dataset at https://www.cepu.gov.hk/en/PRFS/ppr-reports.html.

## 3 Results

Among the 98 participants recruited to the Phase I study, we received responses from 84 (86%) individuals. These participants included 18 patients, 20 carers, 22 professionals and 24 community members (Table 2). Most patients were women (61%) and were aged ≥ 60 years (76%), with advanced COPD (50%) or CKD (44%). The mean time since diagnosis was 15.2 years. Most carers were women (60%) and retired (84%), with the largest age group between 40 and 59 years (45%). Furthermore, 52% of carers were employed, and 56% had a monthly household income below HKD 10,000 (approximately USD 1280). They had engaged in caregiving for a mean of 10.7 years. Advanced CKD (50%) was the most common condition among care recipients. Most carers were children of the care recipients (60%). Among both the patients and carers, 24%–44% received support services from the Hospital Authority, the Social Welfare Department, or an NGO.

**Table 2:** Characteristics of Phase II participants.

Characteristics	Total (*N* = 84)	Patients (*n* = 18)	Carers (*n* = 20)	Professionals (*n* = 22)	Community members (*n* = 24)
Males	25 (30%)	7 (39%)	8 (40%)	6 (27%)	4 (17%)
Age (years)					
18–29	16 (19%)	0 (0%)	2 (10%)	5 (23%)	9 (38%)
30–39	16 (19%)	0 (0%)	3 (15%)	4 (18%)	9 (38%)
40–49	12 (14%)	0 (0%)	4 (20%)	5 (23%)	3 (13%)
50–59	16 (19%)	4 (22%)	5 (25%)	7 (32%)	0 (0%)
60–69	15 (18%)	7 (39%)	5 (25%)	1 (5%)	2 (8%)
≥ 70	9 (11%)	7 (39%)	1 (5%)	0 (0%)	1 (4%)
Educational level					
Primary or below	8 (10%)	8 (44%)	0 (0%)	0 (0%)	0 (0%)
Secondary	22 (26%)	8 (44%)	10 (50%)	2 (9%)	2 (8%)
Tertiary	28 (33%)	1 (6%)	10 (50%)	8 (36%)	9 (38%)
Postgraduate	26 (31%)	1 (6%)	0 (0%)	12 (55%)	13 (54%)
Married	25 (30%)	13 (72%)	12 (60%)	—	—
Employed	51 (61%)	2 (11%)	11 (55%)	22 (100%)	16 (67%)
Household monthly income (HKD)					
< 10,000	17 (20%)	9 (50%)	8 (40%)	—	—
10,000–29,000	8 (10%)	4 (22%)	4 (20%)	—	—
30,000–49,000	5 (6%)	2 (11%)	3 (15%)	—	—
≥ 50,000	5 (6%)	2 (11%)	3 (15%)	—	—
Diagnosis of the care recipient					
Chronic obstructive pulmonary disease	—	9 (50%)	5 (25%)	—	—
Chronic renal failure	—	8 (44%)	10 (50%)	—	—
Heart failure	—	1 (6%)	5 (25%)	—	—
Mean time since diagnosis or time engaged in caregiving/profession (years)	—	15.22	10.72	16.68	—

*Note:* (—) means not applicable.

The majority of the professionals were nurses (50%) or social workers (38%), with a mean of 15.6 years of experience. Furthermore, 58% of the professionals reported being family carers. Of the community members, 33% were from local tertiary institutions and 25% from NGOs. The largest age group was 70 years or older (40%).

**Figure 1:**
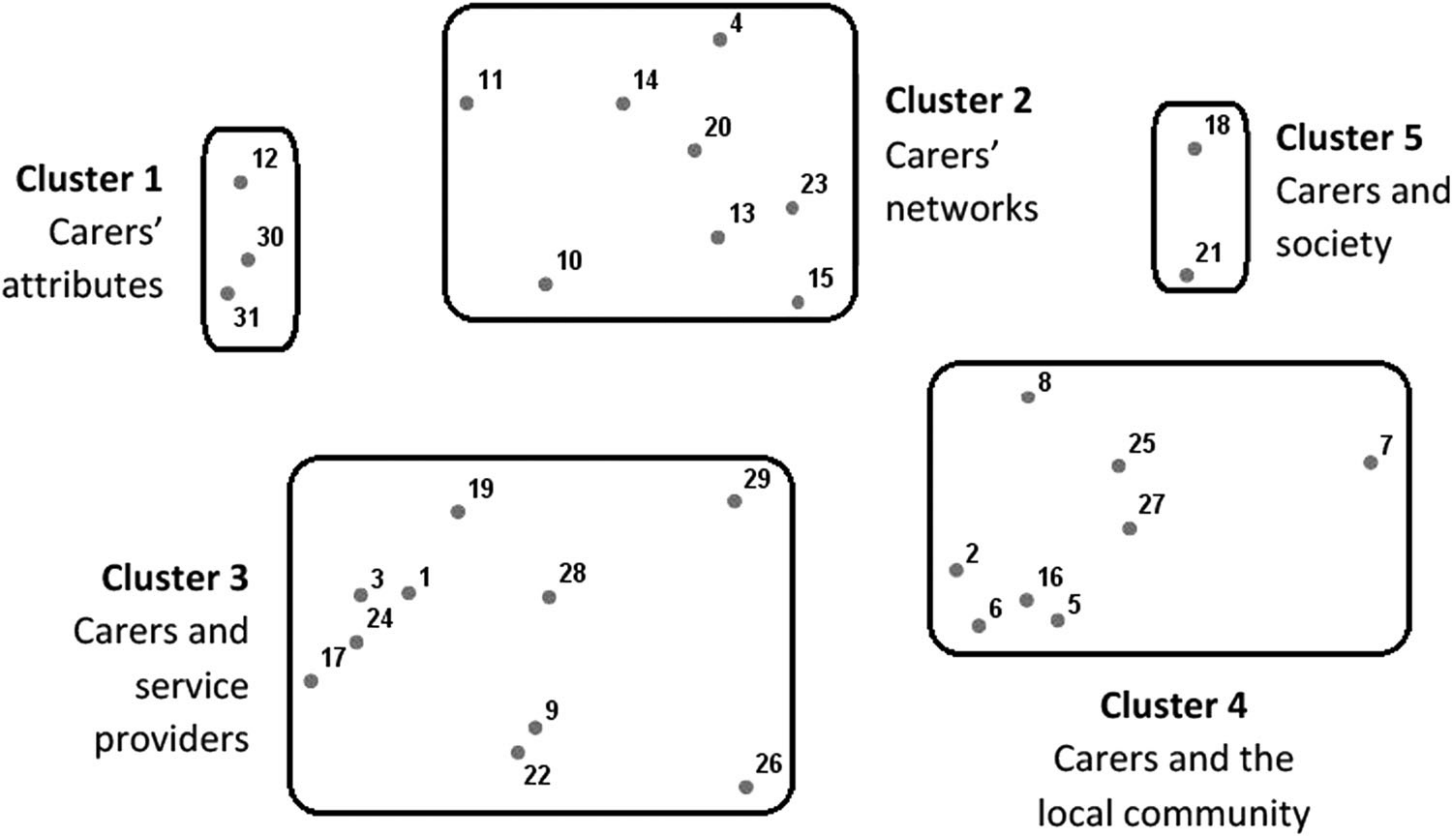
Concept map of different dimensions of social capital. The 31 points represent individual statements. Based on hierarchical cluster analysis results, a 5‐cluster solution was applied to the concept map, with each cluster including 2–10 statements. Goodness‐of‐fitness indices: Kruskal's stress = 0.146 and RSQ = 0.944.

A two‐dimensional final MDS solution was chosen (Figure 1), where the Kruskal's stress and RSQ values were 0.146 and 0.944, respectively, indicating a good fit of the point map for the data. Based on the HCA results, a 5‐cluster solution was applied to the point map. The five clusters included 2–10 statements each and were named as follows: (1) carers' attributes, (2) carers' networks, (3) carers and service providers, (4) carers and the local community, and (5) carers and society.

### 3.1 Cluster 1: Carers' Attributes

This cluster comprised three statements related to the attributes of carers that may facilitate caregiving (Table 3), including positive interaction with the care recipient (mean importance rating = 6.37), a sense of responsibility (6.26) and readiness to accept support (6.24), all of which were bonding social capital. This cluster achieved the second‐highest overall mean importance rating (6.29).

**Table 3:** Importance ratings of statements in the five clusters.

Statements	Means of importance ratings# (standard deviations)
Cluster 1: Carers' attributes	6.29 (0.63)
30. Interactions between patients and carers (Bonding capital)	6.37 (0.76)
12. Sense of responsibility (Bonding capital)	6.26 (0.92)
31. Readiness to accept support (Bonding capital)	6.24 (0.80)
Cluster 2: Carers' networks	6.18 (0.68)
23. Respite care (Bridging capital)	6.40 (0.79)
13. Venting space (Bridging capital)	6.39 (0.79)
10. Caregiving information (Bridging capital)	6.36 (0.91)
11. Support from other family members (Bonding capital)	6.31 (0.93)
15. Reliable, effective communication channels (Bridging capital)	6.27 (0.72)
14. Mutual support of carers (Bonding capital)	6.19 (0.81)
20. Social networks of carers (Bonding capital)	5.93 (0.99)
4. Mutual support of neighbours (Bonding capital)	5.60 (1.24)
Cluster 3: Carers and service providers	6.34 (0.65)
17. Optimising treatments for patients (Bridging capital)	6.60 (0.68)
22. Healthcare professionals' attitude (Bridging capital)	6.49 (0.75)
9. Community‐based healthcare (Bridging capital)	6.46 (0.68)
28. Patient transportation services (Bridging capital)	6.44 (0.88)
19. Financial and material support (Bridging capital)	6.40 (0.81)
24. Timely transition to institutional care (Bridging capital)	6.31 (1.10)
3. Discharge support (Bridging capital)	6.29 (0.99)
1. Home‐based support (Bridging capital)	6.21 (1.25)
26. Healthcare professionals' knowledge of social care (Bridging capital)	6.18 (0.97)
29. Carer‐centred care approach (Bridging capital)	6.04 (1.07)
Cluster 4: Carers and the local community	6.13 (0.76)
8. Increasing resources for carer services (Linking capital)	6.32 (0.84)
2. Need‐oriented service design (Bridging capital)	6.27 (0.94)
16. Coordinating carer services (Linking capital)	6.24 (0.80)
27. Advocacy work (Bridging capital)	6.14 (0.79)
6. Reaching out proactively (Bridging capital)	6.11 (1.21)
7. Researching carer situations (Linking capital)	6.04 (0.87)
5. Referral by social workers (Bridging capital)	6.01 (0.99)
25. Trained volunteer workforce (Bonding capital)	5.90 (1.19)
Cluster 5: Carers and society	6.21 (0.63)
18. Flexible working arrangements (Linking capital)	6.35 (0.77)
21. Inclusive public spaces (Bridging capital)	6.07 (0.79)

^#^ The importance of each statement was rated over the range from 1 (the least important) to 7 (the most important) and is presented as mean (SD), whereas the mean ratings for the clusters are calculated by averaging the consisting items' ratings.

### 3.2 Cluster 2: Carers' Networks

This cluster included eight statements related to various sources and types of support within the personal networks of carers (Table 3). These sources included their family members (6.31), friends (5.93), neighbours (5.60), other carers (6.19) and reliable communication channels (e.g., social media; 6.27). The types of support identified in this cluster included caregiving information (6.36), psychological support (6.39) and respite care (6.40), with four classified as bridging capital and four as bonding capital. The overall mean importance rating of this cluster was 6.18.

### 3.3 Cluster 3: Carers and Service Providers

This cluster comprised 10 statements related to the types and qualities of health and social care services (Table 3). In terms of healthcare, optimal treatments for the care recipient (6.60) and accessibility of services were key concerns. Community‐based service provision (6.46) and transportation for consultations (6.44) were key aspects of the desired healthcare services. Health professionals' knowledge about social care (6.18) and caring attitudes (6.49) could facilitate and motivate caregiving. In terms of social care, carer support in the form of home‐based care (6.21), discharge support (6.29), timely institutional care (6.31), and financial and material assistance (6.40) were beneficial. This support could be provided in a carer‐centred approach (6.04). All 10 statements served as bridging capital. This cluster had the highest overall mean importance rating (6.34).

### 3.4 Cluster 4: Carers and the Local Community

This cluster contained eight statements (four bridging capital, three linking capital and one bonding capital) related to support available in the community where the carers resided (Table 3). Two key sources were identified: social workers (6.01) and volunteers (5.90). Several attributes of the support provided included reaching out proactively (6.11) and focusing on needs (6.27). Additional input regarding coordination (6.24) and resources (6.32) was warranted to enhance the accessibility of this support. Furthermore, research (6.04) and advocacy (6.14) were identified as crucial activities that reinforce support for carers in the community. The overall mean importance rating of this cluster was 6.13.

### 3.5 Cluster 5: Carers and Society

This cluster comprised two statements (one linking capital and one bridging capital) related to the social context that facilitates caregiving (Table 3), including inclusive public spaces (6.07) and flexible working arrangements (6.35). The overall mean importance rating of this cluster was 6.21.

## 4 Discussion

This study provides insights into the perspectives of various stakeholders on the dimensions of social capital that can support the informal carers of patients with advanced chronic illnesses in the community. In this study, the views of stakeholders, including patients, carers, professionals and community members, were rated and sorted to generate a concept map that highlighted five clusters: (1) carers' attributes, (2) carers' networks, (3) service providers, (4) the local community and (5) society. From a community development perspective, this participation of stakeholders evokes internal resources, fostering community cohesion to solve a shared problem. This, in turn, enhances the sustainability of programmes and policies. These findings may serve as a clear framework for the development of social care programmes and policies to support informal carers [29].

Social support, which refers to the various forms of support received from people, has a significant impact on caregiving, while social capital describes the characteristics of this support [30]. Among the five dimensions of social capital identified in this study, healthcare and social services were deemed the most important. This is a form of structured social capital because it reflects relationships established with institutions in society. In addition, this is a form of bridging social capital because the services are provided across racial, ethnic or religious classes [2]. Healthcare and social services are important in the community because they directly meet the complex needs of patients with chronic diseases, including medical treatments, discharge support and financial assistance [31]. Our findings demonstrated the significance of a person‐centred orientation in these services that proactively involves carers' and tailors' support to their needs [32]. The next most important cluster was the attributes of the carers, which can be regarded as a cognitive form of bonding social capital. Cognitive social capital refers to shared norms, values and beliefs within a community that foster social interactions (Woodlock, 2001). Consistent with previous findings, this study identified the carer–care recipient relationship and resilience (i.e., the ability to remain committed and adapt to changes) as significant attributes that facilitate caregiving [33]. Therefore, some carer support interventions target the coping strategies of carer–care recipient dyads, demonstrating positive effects on patient outcomes [34].

The participants in this study valued the support received from family members, friends, neighbours and other carers. This interpersonal network represents a form of bonding capital, which is a type of social capital characterised by close‐knit relationships, trust and cooperation developed among group members; these bonds are evident through emotional support, love and care [1]. These networks can use human resources to help members within the same community and enhance health and social care sustainability. Our findings concerning individual networks showed intriguing expectations regarding people surrounding carers. These networks can provide various forms of support, ranging from information sharing to respite care. However, due to the complexity of chronic illnesses, proper training is needed to equip these people with the necessary support skills [35]. Conversely, the community network highlights the roles of social workers and volunteers in community‐based support. Our findings indicate certain limitations in this type of support, including suboptimal coordination and resource availability. Despite various community health and social services in Hong Kong, resources for supporting people with chronic diseases in the community, especially younger patients, remain insufficient [36]. This highlights a significant gap in the current system.

Finally, the inclusiveness of society can help carers return to their normal social roles. An inclusive public space, characterised by good accessibility and quality, facilitates the physical activities of both carers and care recipients. Many stakeholders emphasised the importance of employers' understanding. Flexible work arrangements can help carers achieve a balance between caregiving and their careers [37]. In some countries, paid caregiving leave is provided, and the rights of carer employees are protected by legislation. These examples of structural capital can create a favourable social context for carers.

The final procedure in concept mapping is utilisation. The research team shared a summary of findings with all participants and presented the concept map at two public seminars, collecting feedback to formulate recommendations. Based on the study findings and the feedback received, the research team formulated several recommendations. First, the five identified clusters illustrate the potential of social capital for creating layers of support for informal carers (Figure 1). Social care programmes and policies can be developed by leveraging the strengths and addressing the weaknesses of each layer. For example, peers can be mobilised to support carers, but it is essential to provide them with appropriate training. In addition, because social capital is based on interactions among social groups, programmes and policies should aim to strengthen the cohesion among these layers. Some communication channels (e.g., social media groups and advertising campaigns) may help facilitate information sharing across layers. Further research may help those at the macro levels (e.g., service providers and the government) understand the needs of various stakeholders.

### 4.1 Implications for Policy and Practice

Evidence indicates that the informal carers of patients with advanced chronic illness encounter numerous challenges, highlighting the need for effective interventions. Given the high ratings from the participants for healthcare and social services; positive interactions with care recipients; support from family members, friends, neighbours and other carers; inclusive public spaces; and flexible working arrangements, these factors should be prioritised when designing interventions for supporting informal carers. Furthermore, while this study was conducted within a specific cultural context, the identified dimensions of social capital provide a flexible framework that can be adapted to other cultural settings. To apply this framework in different contexts, it is essential to engage local stakeholders to develop culturally tailored interventions.

### 4.2 Implications for further Research

Future studies could use other methodologies, such as ethnography, to observe these latent characteristics of the community. In addition, because this study highlighted the perspectives of various stakeholders, there might be differing views across subgroups. Therefore, a subgroup analysis might be performed in future studies. Similarly, additional research on specific types of carers, such as older and younger carers, is warranted, given their unique needs.

### 4.3 Strengths and Limitations

This study addressed a knowledge gap regarding the evidence required for developing interventions supporting informal carers of patients with advanced chronic illnesses in Hong Kong. Using a concept mapping methodology, it provided rich insights into the perspectives of stakeholders involved in such care. However, this study has several limitations that warrant attention. First, this study used a purposive and snowball sampling method, which may have introduced recruitment bias. Although this technique is commonly used in qualitative research to obtain in‐depth data about a phenomenon, it may result in the omission of participants with specific characteristics. For example, participants are more likely to be drawn from relatively active or resource‐rich communities, potentially under‐representing marginalised or less connected populations. In this study, reaching the carers of highly dependent patients, such as those with HF, was challenging due to practical and emotional concerns. Thus, palliative care professionals were invited to supply their views on the needs of these carers. Future research should consider employing more diversified recruitment strategies, such as partnering with community organisations, healthcare institutions and social service agencies to actively reach and engage participants from varied socio‐economic, cultural and demographic backgrounds. Second, concept mapping research relies on the individual accounts of a phenomenon. Some forms of cognitive capital, such as values and norms, are subtle and subconscious and are thus not easily recognised or described. The research team identified this challenge and attempted to elicit this type of capital by incorporating an introduction and probing questions into the interview guide.

## 5 Conclusion

Despite a high caregiving burden experienced by the informal carers of patients with advanced chronic illnesses, various dimensions of social capital can provide adequate community support to them. Key dimensions include the healthcare system and social services, supportive carer–care recipient relationships and resilience, individual networks (e.g., family members, friends, neighbours and other carers), and inclusiveness of the society involving community stakeholders and the employers of carers. These dimensions should be considered when designing interventions to support the carers of patients with advanced chronic illnesses in the community.

## Author Contributions

Conceptualisation and funding acquisition: Marques Shek Nam Ng, Winnie Kwok Wei So, Helen Yue Lai Chan and Carmen Wing Han Chan. Study design and methodology: Marques Shek Nam Ng, Winnie Kwok Wei So, Kai Chow Choi, Wallace Chi Ho Chan, Helen Yue Lai Chan and Carmen Wing Han Chan. Data curation: Marques Shek Nam Ng. Formal analysis: Marques Shek Nam Ng and Kai Chow Choi. Validation and visualisation: Marques Shek Nam Ng. Writing – original draft: Marques Shek Nam Ng. Writing – review and editing: all authors.

## Ethics Statement

This study was approved by the Survey and Behavioural Research Ethics Committee of The Chinese University of Hong Kong (Reference Number: SBRE‐19‐715).

## Conflicts of Interest

The authors declare no conflicts of interest.

## Statistical Checking

K.C.C. is a statistician on the authors' team to check the statistics in this manuscript.

## Protocol Registration

ChiCTR2100044171.

## Data Availability

The data that support the findings of this study are available from the corresponding author upon reasonable request.

## References

[1] R. D. Putnam , R. Leonardi , and R. Y. Nanetti , Making Democracy Work: Civic Traditions in Modern Italy (Princeton University Press, 1993), 1004.

[2] M. Woolcock , “The Place of Social Capital in Understanding Social and Economic Outcome,” Canadian Journal of Policy Research 2, no. 1 (2001): 11–17.

[3] D. Costa , “The Influence of Social Capital on Health Issues Among Transgender and Gender Diverse People: A Rapid Review,” Science & Philosophy 10, no. 2 (2022): 109–131, 10.23756/sp.v10i2.934.

[4] D. Costa , M. Andreucci , N. Ielapi , U. M. Bracale , and R. Serra , “Social Capital in Chronic Disease: An Ethnographic Study,” Science & Philosophy 11, no. 2 (2023): 29–50, 10.23756/sp.v11i2.1308.

[5] L. Coll‐Planas , F. Nyqvist , T. Puig , G. Urrútia , I. Solà , and R. Monteserín , “Social Capital Interventions Targeting Older People and Their Impact on Health: A Systematic Review,” Journal of Epidemiology and Community Health 71, no. 7 (2017): 663–672, 10.1136/jech-2016-20813. 27834223

[6] M. Carradore , “Academic Research Output on Social Capital: A Bibliometric and Visualization Analysis,” International Journal of Sociology and Social Policy 42, no. 13 (2022): 113–134.

[7] A. R. Roth , “Informal Caregiving and Social Capital: A Social Network Perspective,” Research on Aging 42, no. 9–10 (2020): 272–280, 10.1177/0164027520912659. 32342726

[8] Definitions (Family Caregiver Alliance, 2023), https://www.caregiver.org/resource/definitions-0/.

[9] S. D. Lambert , J. V. Levesque , and A. Girgis , “The Impact of Cancer and Chronic Conditions on Caregivers and Family Members,” in Cancer and Chronic Conditions: Addressing the Problem of Multimorbidity in Cancer Patients and Survivors, ed. B. Koczwara (Springer, 2016), 159–202, 10.1007/978-981-10-1844-2_6.

[10] Valuing the Invaluable: Strengthening Supports for Family Caregivers (AARP Public Policy Institute, 2023), https://www.aarp.org/ppi/info-2015/valuing-the-invaluable-2015-update.html?cmp=RDRCT-VALUN_JUN23_015.

[11] W. Fu , J. Li , F. Fang , D. Zhao , W. Hao , and S. Li , “Subjective Burdens Among Informal Caregivers of Critically Ill Patients: A Cross‐Sectional Study in Rural Shandong, China,” BMC Palliative Care 20, no. 1 (2021): 167, 10.1186/s12904-021-00858-4. 34674691 PMC8532289

[12] A. Eberl , “The Effect of Informal Caregiving on Social Capital Investments,” Social Science Research 85 (2020): 102319, 10.1016/j.ssresearch.2019.06.010. 31789185

[13] C. R. May , A. Cummings , M. Myall , et al., “Experiences of Long‐Term Life‐Limiting Conditions Among Patients and Carers: What Can We Learn From a Meta‐Review of Systematic Reviews of Qualitative Studies of Chronic Heart Failure, Chronic Obstructive Pulmonary Disease and Chronic Kidney Disease?,” BMJ Open 6, no. 10 (2016): e011694, 10.1136/bmjopen-2016-011694. PMC5073552 27707824

[14] R. Fu , H. Noguchi , A. Kawamura , H. Takahashi , and N. Tamiya , “Spillover Effect of Japanese Long‐Term Care Insurance as an Employment Promotion Policy for Family Caregivers,” Journal of Health Economics 56 (2017): 103–112, 10.1016/j.jhealeco.2017.09.011. 29040896

[15] Carers Action Plan *2018–2020* (Department of Health and Social Care, 2018), https://assets.publishing.service.gov.uk/government/uploads/system/uploads/attachment_data/file/713781/carers-action-plan-2018-2020.pdf.

[16] The Profile of Persons With Disabilities and Chronic Diseases in Hong Kong and Characteristics of Their Carers (Census and Statistics Department, 2022), https://www.censtatd.gov.hk/tc/EIndexbySubject.html?scode=380&pcode=FA100059.

[17] Policy Support to Carers in Selected Places (Legislative Council Secretariat, 2020), https://www.legco.gov.hk/research-publications/english/1920rt07-policy-support-to-carers-in-selected-places-20200309-e.pdf.

[18] W. M. K. Trochim , “An Introduction to Concept Mapping for Planning and Evaluation,” Evaluation and Program Planning 12, no. 1 (1989): 1–16, 10.1016/0149-7189(89)90016-5.

[19] C. W. Chan , K. C. Choi , W. K. So , and H. Y. Chan , “Concept Mapping in Palliative Medicine Research,” Annals of Palliative Medicine 1, no. 2 (2012): 179–181, http://apm.amegroups.com/article/view/1042. 25841478 10.3978/j.issn.2224-5820.2012.07.01

[20] W. Trochim and M. Kane , “Concept Mapping: An Introduction to Structured Conceptualization in Health Care,” International Journal for Quality in Health Care 17, no. 3 (2005): 187–191, 10.1093/intqhc/mzi038. 15872026

[21] J. G. Burke , P. O'Campo , G. L. Peak , A. C. Gielen , K. A. McDonnell , and W. M. K. Trochim , “An Introduction to Concept Mapping as a Participatory Public Health Research Method,” Qualitative Health Research 15, no. 10 (2005): 1392–1410, 10.1177/1049732305278876. 16263919

[22] C. W. H. Chan , K. C. Choi , H. Y. L. Chan , et al., “Unfolding and Displaying the Influencing Factors of Advance Directives From the Stakeholder's Perspective: A Concept Mapping Approach,” Journal of Advanced Nursing 75, no. 7 (2019): 1549–1562, 10.1111/jan.14017. 30950533

[23] K. M. Chow , C. W. H. Chan , K. C. Choi , I. D. White , K. Y. Siu , and W. H. Sin , “A Practice Model of Sexuality Nursing Care: A Concept Mapping Approach,” Supportive Care in Cancer 29 (2021): 1663–1673, 10.1007/s00520-020-05660-1. 32767106

[24] M. S. N. Ng , W. K. W. So , K. C. Choi , W. C. H. Chan , H. Y. L. Chan , and C. W. H. Chan , “Exploring Social Capital for Family Caregivers of Patients With Chronic Organ Failure: Study Protocol for a Concept Mapping Study,” BMJ Open 12, no. 6 (2022): e063691, 10.1136/bmjopen-2022-063691. PMC9207919 35715187

[25] M. S. N. Ng , W. K. W. So , K. C. Choi , et al., “Social Capital for Carers of Patients With Advanced Organ Failure: A Qualitative Exploration of Stakeholders' Perspectives,” BMC Public Health 24, no. 1 (2024): 670, 10.1186/s12889-024-18213-6. 38429719 PMC10908001

[26] S. R. Rosas and M. Kane , “Quality and Rigor of the Concept Mapping Methodology: A Pooled Study Analysis,” Evaluation and Program Planning 35, no. 2 (2012): 236–245, 10.1016/j.evalprogplan.2011.10.003. 22221889

[27] J. P. Donnelly , “A Systematic Review of Concept Mapping Dissertations,” Evaluation and Program Planning 60 (2017): 186–193, 10.1016/j.evalprogplan.2016.08.010. 27693034

[28] B. S. Everitt , S. Landau , M. Leese , and D. Stahl , Cluster Analysis, 5th ed. (Wiley, 2011).

[29] L. A. Anderson and A. Slonim , “Perspectives on the Strategic Uses of Concept Mapping to Address Public Health Challenges,” Evaluation and Program Planning 60 (2017): 194–201, 10.1016/j.evalprogplan.2016.08.011. 27591959

[30] R. Del‐Pino‐Casado , A. Frías‐Osuna , P. A. Palomino‐Moral , M. Ruzafa‐Martínez , and A. J. Ramos‐Morcillo , “Social Support and Subjective Burden in Caregivers of Adults and Older Adults: A Meta‐Analysis,” PLoS One 13, no. 1 (2018): e0189874, 10.1371/journal.pone.0189874. 29293522 PMC5749735

[31] W. Chan , Y. Cao , E. Y. Lu , W. M. Cheung , and H. W. H. Tsang , “Types of Community Support Services and Self‐Efficacy for Continuous Community Living Among Individuals With Disabilities and Caregivers,” International Journal of Environmental Research and Public Health 19, no. 19 (2022): 12976, 10.3390/ijerph191912976. 36232276 PMC9566762

[32] J. S. Marinho , I. B. Batista , R. A. S. Nobre , et al., “Burden, Satisfaction Caregiving, and Family Relations in Informal Caregivers of Older Adults,” Frontiers in Medicine 9 (2022): 1059467, 10.3389/fmed.2022.1059467. 36619643 PMC9813492

[33] E. Cejalvo , M. Martí‐Vilar , C. Merino‐Soto , and M. T. Aguirre‐Morales , “Caregiving Role and Psychosocial and Individual Factors: A Systematic Review,” Healthcare 9, no. 12 (2021): 1690, 10.3390/healthcare9121690. 34946416 PMC8700856

[34] H. G. Buck , A. Stromberg , M. L. Chung , et al., “A Systematic Review of Heart Failure Dyadic Self‐Care Interventions Focusing on Intervention Components, Contexts, and Outcomes,” International Journal of Nursing Studies 77 (2018): 232–242, 10.1016/j.ijnurstu.2017.10.007. 29128777 PMC7059555

[35] G. Carter , C. Monaghan , and O. Santin , “What Is Known From the Existing Literature About Peer Support Interventions for Carers of Individuals Living With Dementia: A Scoping Review,” Health & Social Care in the Community 28, no. 4 (2020): 1134–1151, 10.1111/hsc.12944. 31919974

[36] A. J. He and V. F. Y. Tang , “Integration of Health Services for the Elderly in Asia: A Scoping Review of Hong Kong, Singapore, Malaysia, Indonesia,” Health Policy 125, no. 3 (2021): 351–362, 10.1016/j.healthpol.2020.12.020. 33422336

[37] R. L. Clancy , G. G. Fisher , K. L. Daigle , C. A. Henle , J. McCarthy , and C. A. Fruhauf , “Eldercare and Work Among Informal Caregivers: A Multidisciplinary Review and Recommendations for Future Research,” Journal of Business and Psychology 35 (2019): 9–27, 10.1007/s10869-018-9612-3.

